# Evaluation of comprehensive geriatric assessment in older patients undergoing pacemaker implantation

**DOI:** 10.1186/s12877-020-01685-7

**Published:** 2020-08-12

**Authors:** Andreas W. Schoenenberger, Ian Russi, Benjamin Berte, Vanessa Weberndörfer, Renate Schoenenberger-Berzins, Piotr Chodup, Remo Beeler, Florim Cuculi, Stefan Toggweiler, Richard Kobza

**Affiliations:** 1 Department of Geriatrics, Inselspital, Bern University Hospital, and University of Bern, Bern, Switzerland; 2 Heart Center Lucerne, Luzerner Kantonsspital, 6000 Lucerne 16, Switzerland; 3 Department of Cardiology, University Hospital Zurich, Zurich, Switzerland

**Keywords:** Pacemaker, Geriatric assessment, Charlson comorbidity index

## Abstract

**Background:**

This study evaluated the use of comprehensive geriatric assessment (CGA) in older patients undergoing pacemaker implantation.

**Methods:**

In this prospective cohort, CGA was performed in 197 patients ≥75 years at pacemaker implantation and yearly thereafter. CGA embraced the following domains: cognition, mobility, nutrition, activities of daily living (ADLs), and falls (with or without loss of consciousness). Based on comorbidities, the Charlson comorbidity index (CCI) was calculated. For predictive analysis, logistic regression was used.

**Results:**

During a mean follow-up duration of 2.4 years, the incidence rates of syncope decreased from 0.46 to 0.04 events per year (*p* < 0.001), and that of falls without loss of consciousness from 0.27 to 0.15 (p < 0.001) before vs. after implantation. Sixty-three patients (32.0%) died. Impaired mobility (OR 2.60, 95%CI 1.22–5.54, *p* = 0.013), malnutrition (OR 3.26, 95%CI 1.52–7.01, *p* = 0.002), and a higher CCI (OR per point increase 1.25, 95%CI 1.04–1.50, *p* = 0.019) at baseline were significant predictors of mortality. Among 169 patients who survived for more than 1 year and thus underwent follow-up CGA, CGA domains did not deteriorate during follow-up, except for ADLs. This decline in ADLs during follow-up was the strongest predictor of later nursing home admission (OR 9.29, 95%CI 1.82–47.49, *p* = 0.007). Higher baseline age (OR per year increase 1.10, 95%CI 1.02–1.20, *p* = 0.018) and a higher baseline CCI (OR per point increase 1.32, 95%CI 1.05–1.65, *p* = 0.017) were associated with a decline in ADLs during follow-up.

**Conclusions:**

CGA is useful to detect functional deficits, which are associated with mortality or nursing home admission after pacemaker implantation. The present study seems to support the use of CGA in older patients undergoing pacemaker implantation as functional deficits and falls are amenable to geriatric interventions.

## Background

In industrialized countries, the use of pacemakers in older patients will increase in the future due to epidemiologic and demographic changes [1]. Previous studies have shown that pacemaker implantations have favorable effects on mortality, syncope and quality of life in older patients [2–5]. However, these outcomes reflect only part of important outcomes in older patients, since functional outcomes after cardiologic interventions are similarly important [6, 7]. Moreover, it has been shown that functional limitations prior to interventions are associated with mortality and worse functional outcomes [6, 8–13]. Pre-procedural functional limitations as well as functional outcomes after interventions are usually ascertained using comprehensive geriatric assessment (CGA) [14]. CGA covers important functional domains, such as cognition, mobility, nutrition, activities of daily living, or falls (with or without loss of consciousness) [14].

Though a recent position paper on pacemaker management suggests performing geriatric assessment in older patients, it is only rarely used in clinical routine [1]. This underuse may be partially explained by a lack of good evidence in current literature. To the best of the authors’ knowledge, no previous study evaluated CGA in older patients undergoing pacemaker implantation. Despite an extensive literature search, the authors found only one small study that evaluated cognitive function in 19 patients undergoing pacemaker implantation [15]. The present study therefore aimed to fill this scientific gap and to evaluate CGA in a prospective cohort of older patients undergoing pacemaker implantation.

## Methods

### Study population

Consecutive patients ≥75 years of age undergoing pacemaker implantation and being followed at the Heart Center Lucerne (Lucerne, Switzerland) between March 1st, 2012, and March 31st, 2017, were eligible for this prospective cohort study. The following patients were excluded from the study: first, patients who refused study participation and did not provide a signed informed consent; second, patients in whom baseline assessment was unfeasible due to logistic reasons. Patients with intracardiac cardioverter devices (ICDs) were also excluded. The final study population consisted of all patients in whom pacemaker implantation and baseline examination were performed during the study period. This study complies with the Declaration of Helsinki and was approved by the local ethics committee.

### Baseline evaluation

All participating patients received extensive cardiologic baseline examination according to current guidelines [16]. Patient history was recorded including symptoms, cardiovascular risk factors, medication, prior cardiovascular events, as well as comorbidities. Physical examination included standard parameters such as body weight, height and blood pressure. Based on the number of comorbidities, the Charlson comorbidity index (CCI) was calculated [17].

In addition, all participating patients underwent CGA involving instruments for functional status. CGA consisted of the following validated instruments: Mini Mental State Exam (MMSE) for cognitive function [18], Timed Get Up and Go Test (TUG) for gait function [19], Mini Nutritional Assessment (MNA) for nutritional status [20], Basic Activities of Daily Living (BADL) [21], and Instrumental Activities of Daily Living (IADL) [22]. The number of falls without loss of consciousness and/or syncopes in the preceding 12 months were assessed using standardized questions. The instruments were dichotomized at standard cut points. The latter were defined *from the outset*: MMSE at < 27 points (cognitive impairment) vs. ≥27 points (normal cognitive function), TUG at ≥20 s (mobility impairment) vs. < 20 s (normal gait function), MNA at < 12 points (at risk of malnutrition) vs. ≥12 points (not at risk of malnutrition) [18–20]. If there was one or more activity with a limitation, BADL and IADL were considered abnormal [21, 22]. In addition to the functional assessment, the emotional status was assessed using the 5-item geriatric depression scale (GDS), which was dichotomized at ≥2 points (at risk of depression) vs. < 2 points (not at risk of depression) [23].

### Pacemaker implantation

Pacemaker implantations were performed by five experienced operators according to best clinical practice respecting the current guideline recommendation [16]. Dual chamber pacemakers were implanted in 159 patients, single chamber devices in 33 patients and 5 patients received a biventricular system.

### Follow-up

A routine follow-up was performed yearly at the Heart Center Lucerne in all participants. The study participants were invited by mail and/or phone. In case the participants did not respond to the invitation, the treating general practitioner and/or registered relatives were contacted. During the on-site visit, the pacemaker was interrogated and patients underwent a cardiological examination as well as CGA. CGA consisted of the same instruments that were used at baseline. CGA also assessed the number of falls without loss of consciousness and/or syncopes since the baseline examination or the last follow-up, respectively. In case of death phone interviews were done with the treating general practitioner and/or relatives to ascertain the circumstances. Death and the time of death were crosschecked using the official register of the local authorities.

### Outcomes

Mortality was the main outcome and defined as overall mortality from cardiac or non-cardiac causes. Changes in the incidence of syncope and falls between the pre- and post-implantation period was another outcome. Syncope was defined as fall with a short period of unconsciousness, whereas a drop without loss of consciousness was considered a fall. As a further outcome, we determined the change of functional status between baseline and follow-up comparing the single CGA domains between baseline and follow-up. Based on BADL and IADL, we defined the outcome decline in activities of daily living (decline in ADLs), which corresponded to a deterioration of ≥1 activity in the ability to perform BADL and/or IADL between baseline and follow-up. Regarding the outcome nursing home admission, only new admissions after pacemaker implantation were considered.

### Statistical analysis

First, we descriptively analyzed mortality, syncope and falls without loss of consciousness during follow-up. Second, to compare rates of syncope and falls during follow-up with the pre-implantation period, incidence rates were calculated and compared using the incidence rate ratio (IRR) with its 95% confidence interval (CI). Third, we descriptively analyzed the change of functional status between baseline evaluation and last available follow-up examination among patients who survived for more than 1 year after pacemaker implantation and thus had at least one follow-up CGA. Fourth, in order to compare the functional status during follow-up with baseline, we used logistic regression reporting odds ratios (ORs) with 95% CIs and *p* values. Models were done unadjusted as well as adjusted for age, sex and the CCI. Fifth, we separately analyzed the functional course among surviving patients, who showed a baseline impairment in the respective domain. The rational for this analysis was the high clinical relevance of this subgroup; furthermore, due to the ceiling effect of the assessment instruments, improvements could only be shown for this subgroup. Sixth, we used multivariable logistic regression to identify associations between potentially predictive variables and the outcomes. The potentially predictive variables were selected a priori based on clinical considerations (i.e., age, sex, CCI, and the CGA domains). In the logistic models, the CCI was used as categorized variable with a range from 1 to 9, and the CGA domains were dichotomized at the standard cut points. The area under the receiver operating characteristic curve (AUROC) was used to quantify predictive ability. Finally, we performed sensitivity analyses. For mortality prediction, we performed a sensitivity analysis using a modified CCI without cardiovascular comorbidities, but adding cardiovascular comorbidities (i.e., NYHA class, presence of coronary artery disease, previous myocardial infarction, and heart failure) as single independent variables to the logistic model. For the prediction of nursing home admission, we performed a further sensitivity analysis retaining the deceased patients in the regression model. Data were analyzed using Stata 12.1 (StataCorp LP, College Station, TX, USA).

## Results

### Study population

**Fig. 1:**
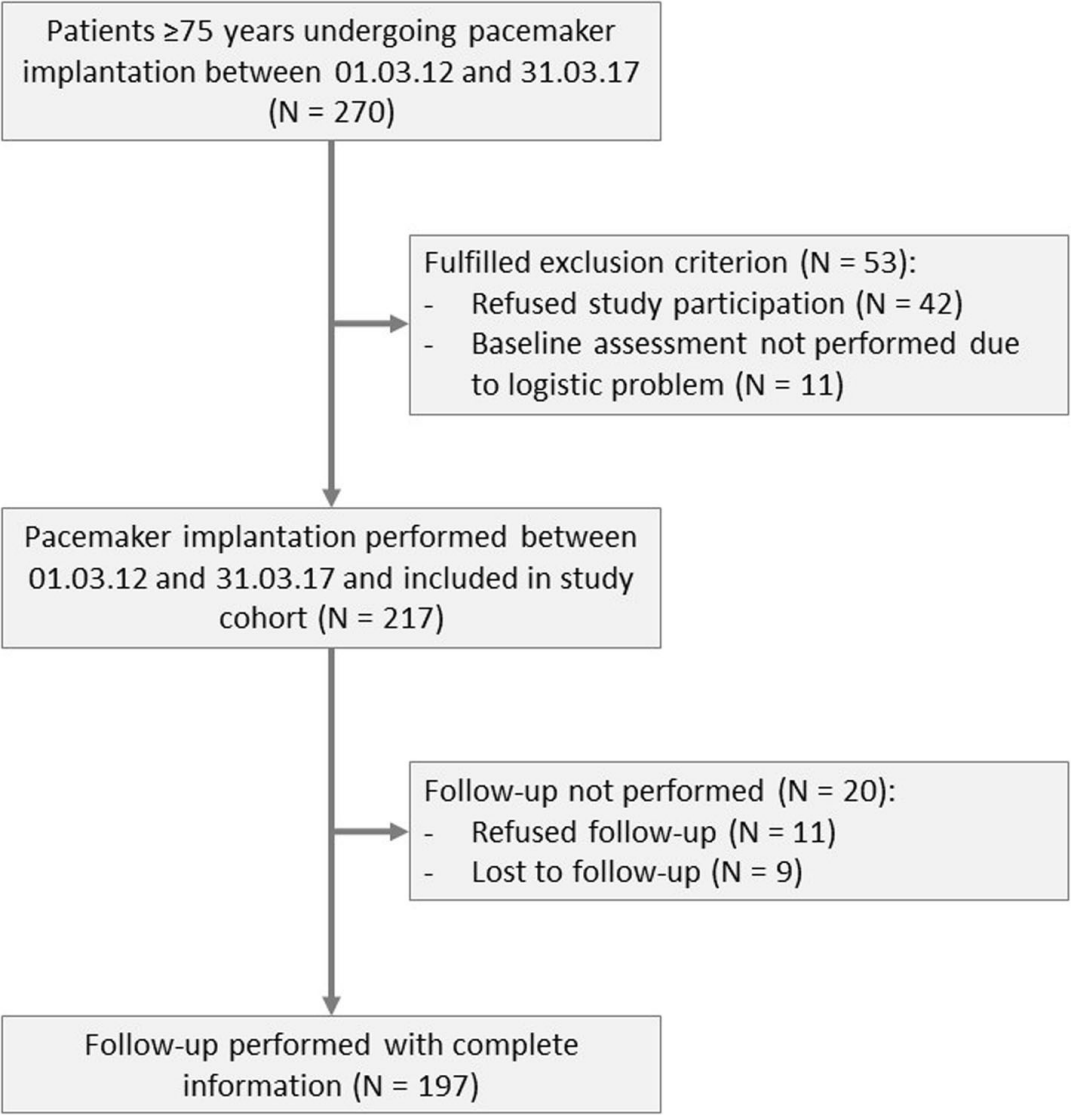
Flow chart

Between March 1st 2012 and March 31st 2017, 270 patients aged 75 years or older underwent pacemaker implantation (Fig. 1). Fifty-three patients were initially excluded: 42 patients refused participation, 11 did not have a complete baseline assessment due to logistic problems. Of 217 eligible patients, 11 (5.1%) refused follow-up examination and 9 (4.1%) were lost to follow-up. The study population finally consisted of 197 patients.

Baseline characteristics are shown in Table 1. Mean age was 82.9 ± 4.9 years (range 75–95 years). Sinus node disease, higher degree atrioventricular block and atrial fibrillation were frequent ECG findings. Accordingly, the most frequent indication for pacemaker implantation was bradycardia (137 patients, 69.5%). Syncope as well as falls without loss of consciousness were frequent events in the 12 months preceding the pacemaker implantation (Table 1). Thirty-seven patients (18.8%) have suffered from severe injuries associated with the syncopal event or fall (e.g., bone fracture in 16 patients, bleeding needing intervention in 10 patients, traumatic brain injury in 8 patients, other serious injury in 3 patients). Many of the patients had functional limitations based on CGA (Table 1).

**Table 1:** Baseline characteristics

Characteristic	All study participants (***N*** = 197)
Age, mean ± SD, years	82.9 ± 4.9
Female sex, n (%)	86 (43.7%)
Body Mass Index, mean ± SD, kg/m2	25.7 ± 4.2
***Cardiovascular risk factors***	
Hypertension, n (%)	155 (78.7%)
Hypercholesterolemia, n (%)	60 (30.5%)
Current smoker, n (%)	17 (8.6%)
Diabetes, n (%)	30 (15.2%)
Positive family history, n (%)	24 (12.2%)
***Medical history***	
Coronary artery disease, n (%)	92 (46.7%)
Previous myocardial infarction, n (%)	16 (8.1%)
Valvular heart disease, n (%)	92 (46.7%)
Hypertensive heart disease, n (%)	40 (26.9%)
Hypertrophic cardiomyopathy, n (%)	24 (12.2%)
Previous stroke, n (%)	25 (12.7%)
Previous syncope within last 12 months, n (%)	90 (45.7%)
Fall without loss of consciousness within last 12 months, n (%)	53 (26.9%)
***Electrocardiogram***	
Sinus node disease, n (%)	49 (24.9%)
Atrioventricular block	
- Second-degree, n (%)	39 (19.8%)
- Third-degree, n (%)	51 (25.9%)
Atrial fibrillation, n (%)	59 (29.9%)
***Comprehensive geriatric assessment***	
Cognitive impairment (MMSE < 27 points), n (%)	84 (42.6%)
Mobility impairment (TUG ≥20 s), n (%)	71 (36.0%)
At risk of malnutrition (MNA < 12 points), n (%)	90 (45.7%)
BADL with ≥1 activity with limitation, n (%)	39 (19.8%)
IADL with ≥1 activity with limitation, n (%)	110 (55.8%)
***Emotional assessment***	
At risk of depression (GDS ≥2 points), n (%)	34 (17.3%)
***Comorbidity burden***	
Charlson comorbidity index ≥3, n (%)	98 (49.8%)

*Abbreviations*: *BADL* basic activities of daily living, *GDS* geriatric depression scale, *IADL* instrumental activities of daily living, *MMSE* mini mental state exam, *MNA* mini nutritional assessment, *TUG* timed get up and go test

### Mortality, syncope and falls in all study participants

During a mean follow-up of 2.4 ± 1.4 years and total observation time of 472 person-years, 63 patients (32.0%) died. Most of these deaths (88.9%) were considered to be of a non-cardiac origin (e.g., general weakness due to age, infectious disease, and/or carcinoma).

During follow-up, 19 syncopes and 71 falls without loss of consciousness occurred. Compared to the pre-implantation period the incidence rate of syncope dramatically decreased from 0.46 to 0.04 events per year (IRR 0.09, 95% CI 0.05–0.15, *p* < 0.001), as did falls without loss of consciousness with a decreasing incidence rate from 0.27 to 0.15 events per year (IRR 0.56, 95% CI 0.39–0.81, *p* < 0.001).

### Functional course

Table 2 shows the changes in functional and emotional status among the 169 patients with available follow-up CGA. Overall, functional and emotional status were well preserved until the last follow-up, with the exception of activities of daily living. BADL and IADL showed a significant deterioration over the study period.

**Table 2:** Functional and emotional course (*N* = 169)

Domain	Baseline	Follow-up	Regression analysis			
			***Unadjusted***		***Adjusted*** ^a^	
	*N (%)*	*N (%)*	*OR (95% CI)* ^b^	*P value* ^b^	*OR (95% CI)* ^b^	*P value* ^b^
***Cognition***						
Cognitive impairment (MMSE < 27 points)	64 (37.9%)	69 (40.8%)	1.13 (0.73–1.75)	0.578	1.15 (0.73–1.81)	0.560
***Mobility***						
Mobility impairment (TUG ≥20 s)	55 (32.5%)	49 (29.0%)	0.85 (0.54–1.36)	0.502	0.85 (0.53–1.37)	0.504
***Activities of daily living***						
BADL with ≥1 activity with limitation	29 (17.2%)	48 (28.4%)	1.92 (1.14–3.23)	0.015	2.18 (1.23–3.88)	0.008
IADL with ≥1 activity with limitation	90 (53.3%)	121 (71.6%)	2.21 (1.41–3.47)	0.001	2.59 (1.57–4.26)	< 0.001
***Nutrition***						
At risk of malnutrition (MNA < 12 points)	71 (42.0%)	60 (35.5%)	0.76 (0.49–1.18)	0.220	0.74 (0.47–1.17)	0.198
***Emotion***						
At risk of depression (GDS ≥2 points)	22 (13.0%)	24 (14.2%)	1.11 (0.59–2.06)	0.751	1.11 (0.59–2.08)	0.749

*Abbreviations*: *BADL* basic activities of daily living, *GDS* geriatric depression scale, *IADL* instrumental activities of daily living, *MMSE* mini mental state exam, *MNA* mini nutritional assessment, *TUG* timed get up and go test

^a^ Adjustment variables include age, sex and Charlson comorbidity index

^b^ Odds ratio (OR) with 95% confidence interval (CI) and *p* value from logistic regression for the comparison of follow-up vs. baseline status

An additional analysis of the subgroup of surviving patients with baseline impairment showed that relevant proportions of these patients improved in their functional and emotional course. Among the 46 surviving patients with cognitive impairment at baseline, 27 patients (58.7%) showed a cognitive improvement, 7 patients (15.2%) remained unchanged, and only 12 patients (26.1%) showed a further deterioration. Among the 37 surviving patients with mobility impairment at baseline, mobility improved in 21 patients (56.8%), remained unchanged in 13 patients (35.1%), and deteriorated in only 3 patients (8.1%). Nutritional status improved in 36 (75.0%) of the 48 surviving patients with risk of malnutrition at baseline, remained unchanged in 9 patients (18.8%), and deteriorated in only 3 patients (6.2%). Regarding BADL and IADL, improvements were found in 12 of 21 patients (57.1%) with BADL limitations and 22 of 70 patients (31.4%) with IADL limitations at baseline; a deterioration was found in 6 patients (28.6%) for BADL and 26 patients (37.1%) for IADL, respectively. Regarding emotional status, an improvement was found in 13 of 16 patients (81.3%), while 3 patients (18.7%) remained stable and no patient deteriorated.

### Predictive analyses

**Fig. 2:**
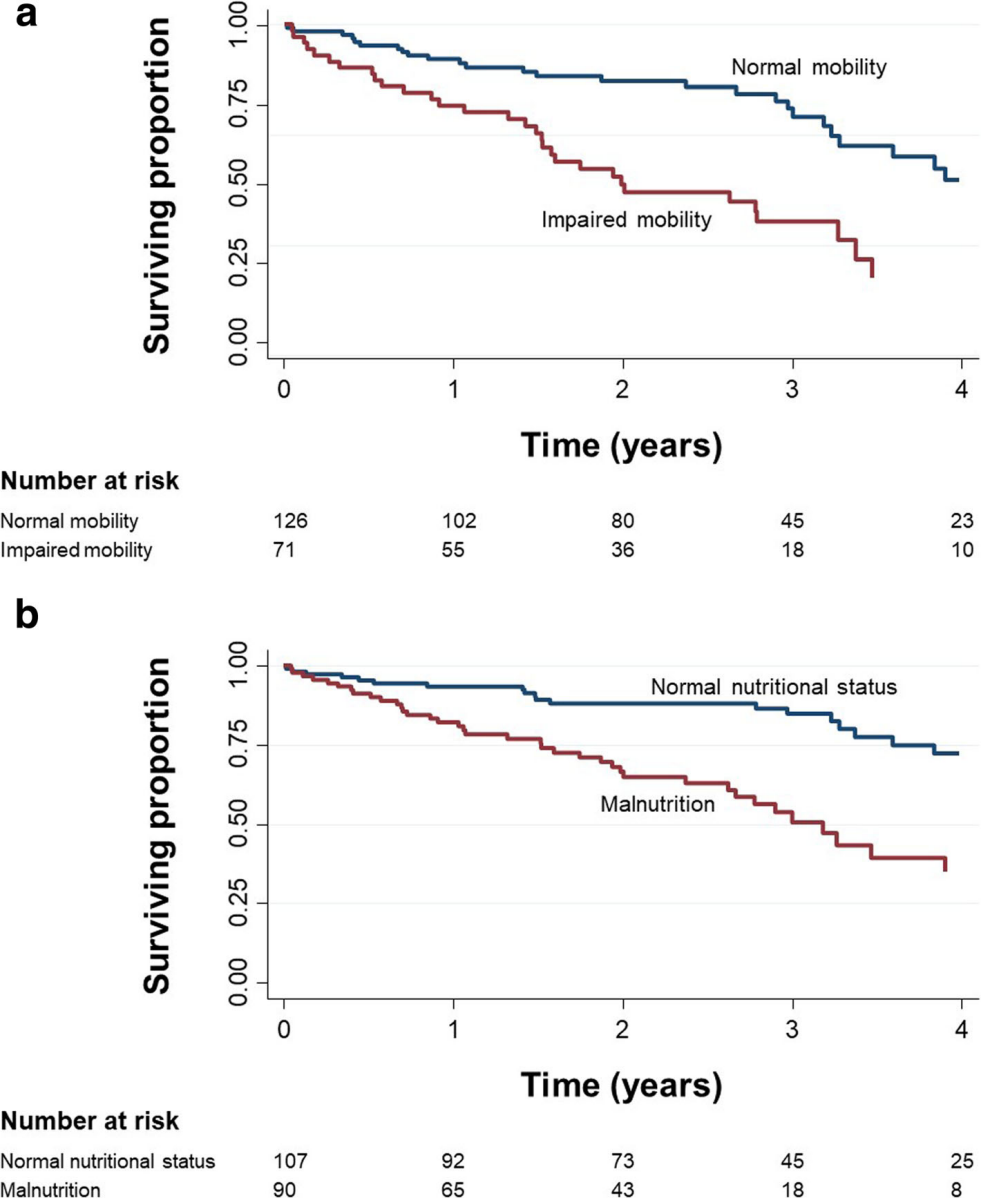
**a** Kaplan-Meier survival estimates stratified by mobility at baseline. **b** Kaplan-Meier survival estimates stratified by nutritional status at baseline

In the predictive analysis, impaired mobility (OR 2.60 [95% CI 1.22–5.54], *p* = 0.013; AUROC 0.63 [95% CI 0.56–0.70]), malnutrition (OR 3.26 [95% CI 1.52–7.01], *p* = 0.002; AUROC 0.65 [95% CI 0.58–0.73]), a higher CCI (OR per point increase 1.25 [95% CI 1.04–1.50], *p* = 0.019; AUROC 0.68 [95% CI 0.60–0.75]), and age (OR per year increase 1.08 [95% CI 1.00–1.16], *p* = 0.049; AUROC 0.62 [95% CI 0.53–0.70]) at baseline were significant predictors of mortality after the pacemaker implantation (Fig. 2). Of the 134 surviving patients, 13 patients (9.7%) were newly admitted to a nursing home. The decline in ADLs from baseline to follow-up was found to be the strongest predictor of later nursing home admission (OR 9.29 [95% CI 1.82–47.49], *p* = 0.007; AUROC 0.72 [95% CI 0.61–0.84]), whereas the baseline variables in the model (i.e., age, sex, CCI, and CGA domains) were not predictive of the outcome. In a logistic regression evaluating baseline factors associated with the decline in ADLs, significant associations were found for higher baseline age (OR per year increase 1.10 [95% CI 1.02–1.20], *p* = 0.018; AUROC 0.58 [95% CI 0.49–0.68]) and a higher CCI (OR per point increase 1.32 [95% CI 1.05–1.65], *p* = 0.017; AUROC 0.59 [95% CI 0.49–0.68]).

### Sensitivity analyses

Using cardiovascular comorbidities as single independent predictors in the model for mortality prediction, impaired mobility, malnutrition, and the modified CCI were unvaried the significant predictors of mortality after pacemaker implantation, whereas the cardiovascular comorbidities did not significantly predict mortality. Including the deceased patients in the analysis for the prediction of nursing home admission, the decline in ADLs from baseline to follow-up remained the strongest predictor of later nursing home admission (OR 9.92 [95% CI 2.12–46.48], *p* = 0.004).

## Discussion

The results from this prospective cohort show that, apart from higher age and higher comorbidity burden, impaired mobility and malnutrition were significantly associated with mortality after pacemaker implantation. Mortality in this cohort was high with nearly one of three patients dying during the relatively short mean follow-up duration of little more than 2 years. Pacemaker implantation not only improved the occurrence of syncope, but also led to a highly relevant decrease in falls without loss of consciousness. Our study further shows that functional status remains stable in surviving patients after pacemaker implantation except for activities of daily living, which deteriorated during follow-up. The decline in ADLs was the most important factor being significantly associated with nursing home admission during follow-up.

Mortality after pacemaker implantation was high in our cohort, but comparable to previous studies involving similar patients [2–5, 24, 25]. Mortality, syncope and quality of life of elderly patients undergoing pacemaker implantation have been well investigated in these previous studies, but to our knowledge this is the first study evaluating CGA in older patients undergoing pacemaker implantation. This study documents the importance of additionally assessing falls without loss of consciousness and the functional course after pacemaker implantation using standardized CGA instruments. This study therefore adds to a growing evidence from other surgical or interventional procedures that CGA is important for the assessment of functional outcomes as well as for outcome prediction [6–13, 26].

This study has clinical implications. It demonstrated that older malnourished patients with impaired mobility have a greater risk of dying after a pacemaker implantation. CGA is suited to detect these functional deficits. Recent systematic analyses of randomized controlled studies have shown that CGA and subsequent interventions based on CGA improve prognosis and decrease nursing home admissions among older patients [27, 28]. This study shows that a decline in ADLs during follow-up increases the risk of nursing home admission. CGA during follow-up detects this deterioration, which may prompt interventions aiming at reversing the decline in ADLs. Therefore, the present study seems to support the use of CGA in older patients undergoing pacemaker implantation.

This study also shows that a relevant proportion of patients with functional limitations at baseline recover from these limitations after pacemaker implantation. We presume that in some patients the functional limitations are the consequence of hemodynamic compromises due to the cardio-electric dysfunction leading to pacemaker implantation. This study therefore underlines the importance of performing pacemaker implantation even in the oldest old.

This study has limitations. First, the findings of this study are based on data from a single center. Therefore, generalizability of the findings of the present study is limited. Second, though the sample size was adequate for most analyses, the number of study participants and endpoints was probably too low for certain analyses. It is conceivable that some of the non-significant results might be the consequence of a type II error. Third, 9 of the 217 patients (4.1%) included in the study cohort were lost to follow-up without information on mortality, which may have biased our follow-up results to a certain extent. Fourth, as functional course could only be analyzed for patients, who did not die during the first year of follow-up and thus underwent follow-up CGA, the results of functional course might be biased to some extent. However, we performed a sensitivity analysis for the prediction of nursing home admission including the deceased patients, which showed no relevant change of the predicting factors. Finally, this study only documents associations of CGA with important outcomes, but, due to the nature of a cohort study, fails to show whether or not CGA ultimately improved patient outcomes. Therefore, this study also has implications for research. Randomized controlled trials are urgently needed to determine the effect of CGA on outcomes in specific clinical conditions, such as cardiologic, surgical or oncologic procedures [26].

## Conclusion

This study shows that CGA is a useful tool to detect functional deficits, which are associated with mortality or nursing home admission after pacemaker implantation. The present study seems to support the use of CGA in older patients undergoing pacemaker implantation as functional deficits and falls are amenable to geriatric interventions.

## Data Availability

The datasets used and/or analysed during the current study are available from the corresponding author on reasonable request.

## Abbreviations

ADLs: Activities of daily living
BADL: Basic activities of daily living
CCI: Charlson comorbidity index
CGA: Comprehensive geriatric assessment
CI: Confidence interval
GDS: Geriatric depression scale
IADL: Instrumental activities of daily living
IRR: Incidence rate ratio
MMSE: Mini mental state exam
MNA: Mini nutritional assessment
OR: Odds ratio
TUG: Timed get up and go test
